# “Impact of and response to increased tuberculosis prevalence among Syrian refugees compared with Jordanian tuberculosis prevalence: case study of a tuberculosis public health strategy”

**DOI:** 10.1186/s13031-015-0044-7

**Published:** 2015-05-18

**Authors:** Susan T. Cookson, Hiba Abaza, Kevin R. Clarke, Ann Burton, Nadia A. Sabrah, Khaled A. Rumman, Nedal Odeh, Marwan Naoum

**Affiliations:** US Centers for Disease Control and Prevention, Atlanta, GA USA; International Organization for Migration, Amman, Jordan; United Nations High Commissioner for Refugees, Amman, Jordan; Hashemite Kingdom of Jordan National TB Program, Amman, Jordan

**Keywords:** Tuberculosis, TB control program, Refugees, Syrian crisis, Jordan

## Abstract

**Introduction:**

By the summer of 2014, the Syrian crisis resulted in a regional humanitarian emergency with 2.9 million refugees, including 608,000 in Jordan. These refugees access United Nations High Commissioner for Refugees (UNHCR)-sponsored clinics or Jordan Ministry of Health clinics, including tuberculosis diagnosis and treatment. Tuberculosis care in Syria has deteriorated with destroyed health infrastructure and drug supply chain. Syrian refugees may have undiagnosed tuberculosis; therefore, the UNHCR, the International Organization for Migration (IOM), the National Tuberculosis Program (NTP), and the Centers for Disease Control and Prevention developed the *Public Health Strategy for Tuberculosis among Syrian Refugees in Jordan*. This case study presents that strategy, its impact, and recommendations for other neighboring countries.

**Case description:**

UNHCR determined that World Health Organization (WHO) criteria for implementing a tuberculosis program in an emergency were met for the Syrian refugees in Jordan. Jordan NTP assessed their tuberculosis program and found that access to Syrian refugees was the one component of their program missing. Therefore, a strategy for tuberculosis control among Syrian refugees was developed. Since that development through work with IOM, UNHCR, and NTP, tuberculosis case detection among Syrian refugees is almost 40 % greater (74 cases/12 months or 1.01/100,000 monthly through June 2014 vs. 56 cases/16 months or 0.73/100,000 monthly through June 2013) using estimated population figures; more than two fold the 2012 Jordan tuberculosis incidence. Additionally, the WHO objective of curing ≥85 % of newly identified infectious tuberculosis cases was met among Syrian refugees.

**Discussion and evaluation:**

Tuberculosis (TB) rates among displaced persons are high, but increased detection is possible. High TB rates were found among Syrian refugees through active screening and will probably persist as the Syrian crisis continues. Active screening can detect tuberculosis early and reduce risk for transmission. However, this strategy needs sustainable funding to continue and all activities have not been realized.

**Conclusions:**

Initial assessment found that tuberculosis among Syrian refugees was at a high incidence rate. Through partnership, a cohesive Jordanian tuberculosis strategy was developed for Syrian refugees and it has potential to inform treatment and control efforts for other regional countries impacted by the Syrian crisis.

## Background

From March 2011 through July 2014, the Syrian crisis has resulted in a regional humanitarian emergency with 2.9 million Syrian refugees, including 608,000 in Jordan [1]. In Jordan, only 15 % of Syrian refugees are residing in camps; the majority of refugees are integrated into host communities (non-camp refugees). Host community-based registered and awaiting registration refugees are entitled to access Hashemite Kingdom of Jordan Ministry of Health clinics and United Nations High Commissioner for Refugees (UNHCR)-sponsored clinics, respectively. These and camp-based refugees have access to governmental chest clinics.

Prior to March 2011, Syria was making public health gains in tuberculosis (TB) prevention, reducing annual TB prevalence from 85 TB cases per 100,000 persons in 1990 to 23 per 100,000 in 2011 [2]. Tuberculosis care in Syria was integrated in the healthcare system nationwide with specialty TB treatment facilities located in each governorate, including Aleppo and Homs, areas badly destroyed by the conflict. As the conflict escalated, health infrastructure has been destroyed, drug supply chains have been interrupted, and healthcare workers have fled [3, 4]; all negatively impacting TB diagnosis and treatment efforts.

The Syrian refugee influx in Jordan resulted in TB patients seeking treatment at camp and non-camp clinics, placing pressure on Jordan National TB Program (NTP) resources. From March 2012 through June 2013, the International Organization for Migration (IOM) and the Jordan NTP confirmed 56 cases of TB among Syrian refugees, including 3 multidrug-resistant (MDR)-TB cases. Therefore, with the technical assistance of the US Centers for Disease Control and Prevention (CDC), the national public health strategy was revised to target TB reduction in this vulnerable population through development of the *Public Health Strategy for Tuberculosis among Syrian Refugees in Jordan* [5] in July 2013 by the NTP, UNHCR and IOM with approval from the World Health Organization-Jordan. This case study presents the strategy and the impact of that strategy as well as recommendations for other neighboring countries.

## Case description

The Jordan NTP reached the Millennium Development Goal for TB reduction in 2011 and was preparing to shift to TB elimination. However, TB elimination planning has been postponed because of the Syrian crisis and refugee influx.

### Criteria for implementing TB control programs

A poorly implemented TB control program can potentially prolong the time a patient is infectious resulting in greater transmission of TB. Therefore, the World Health Organization (WHO) has outlined essential pre-requisite criteria to meet before implementing TB programs in emergencies [6].

These criteria include

1. Population data indicate TB is an important health problem
2. Acute phase of the emergency is over
3. Population is stable for at least 6 months
4. Basic needs are met
5. Essential health care services and drugs for common illnesses are available, and primary care health services are accessible so that patients with suspected TB can be identified, investigated, and referred, if necessary.

In June 2013, IOM and CDC met with UNHCR and WHO-Jordan to go point-by-point over these criteria to determine if it was appropriate to offer TB care and control to Syrian refugees. For criterion 1, the TB prevalence in Syria was three-fold higher than in Jordan for 2011; 23 per 100,000 persons in Syria compared with 7.7 per 100,000 persons for Jordan [2]. For criterion 2, “although new arrivals continue in large numbers, mortality estimates among refugees in Za’atri camp are well below emergency levels and the health response has been increasingly focused on chronic disease management” [5]. For criterion 3, the Syrian crisis was in its third year at the time of the convening of the partners. It was expected that the population would remain in Jordan for the unforeseen future, probably much longer than 6 months. For criteria 4 and 5, the Government of Jordan, UNHCR, and other humanitarian actors have established camps for the Syrian refugees as well as services and support for non-camp refugees; these include access to basic services, including food, and to essential healthcare services. Therefore, these WHO criteria were met in Jordan.

#### Public health strategy

Despite challenges in providing stability and essential services before implementing TB control in refugee settings, the strength of the Jordan TB control program offered a unique opportunity to improve refugee health, prevent new TB cases through early detection and reduced transmission, and minimize the emergence of MDR-TB. In June 2013, IOM and CDC met with Jordan NTP to develop a stepwise approach to ‘reduce susceptible and resistant tuberculosis transmission, morbidity, and mortality among Syrian refugees residing in Jordan’ [5]. The idea was that detection and treatment of TB cases among Syrian refugees would continue to occur through partnership of IOM and NTP, respectively, but with a more systematic approach.

Before development of the strategy, Jordan NTP assessed their TB care and control program to determine if they could adequately provide TB care and control for the Syrian refugees. They examined their i) lead agency and coordination; ii) policies, including the provision of directly observed therapy short-course (DOTS) [7]; iii) availability of human resources for the additional patient influx from Syria; iv) access to the Syrian refugees; v) type (microscopic, culture and drug-susceptibility testing capacity), location and quality assurance of diagnostic laboratory facilities; vi) treatment regimens; vii) drug procurement and storage; and viii) follow-up procedures. All of these were found to be adequate except for the iv) access to the new influx of Syrian refugees. Therefore, Jordan NTP wanted a public health strategy developed to include Syrian refugees.

The strategic objectives were [5]:

1. Increase TB screening among Syrian refugees initially processed in UNHCR camps, or among those already residing in camp or non-camp settings, by camp- or facility-based health assessments, respectively
2. Increase TB diagnosis among Syrian refugees who were patients with suspected TB. In addition, identified refugees already with TB diagnosis with or without receiving anti-TB treatment before arrival in Jordan. IOM and/or NTP assessed these individuals for the length of time without treatment, provided additional testing, if needed, and then restarted on treatment, if indicated. IOM with other health partners conducted symptomatic TB screening of Syrian refugees following the NTP diagnostic procedure and all suspected cases received chest radiographs. If radiographs were abnormal, three acid-fast bacilli (AFB) smears and culture were performed to confirm diagnosis. If the AFB results were negative and there was no improvement after receiving non-TB antibiotics, the diagnosis was based on radiograph finding and medical officer’s judgment.
3. Maximize treatment success among Syrian refugees for those remaining in Jordan or moving to other countries. Four activities were undertaken: i) DOTS was instituted using Jordan NTP case management forms; ii) contingency plans for movement to other countries for treatment were developed; however, refugees were counseled to complete treatment in Jordan and to sign a modified World Care Counsel patient charter found in the CDC’s Evaluation Tool for Tuberculosis Programs in Resource-limited, Refugee and Post-Conflict Settings, version 2 [8]. For consenting patients, WHO and IOM agreed to assist with transfers. At time of treatment initiation, the refugee was given a DOTS card updated with regimen administered, doses given, and pertinent laboratory results, which s/he could take to other treatment locations, either within Jordan or other countries; iii) psychosocial support was provided by the Jordanian Anti TB Association (JATA) to increase treatment success, alleviate fears, and reduce stigma. TB patients often deal with stigma [9]; in addition, refugees have often witnessed traumatic events and struggle with the consequences of displacement adding to their need for psychosocial support; and iv) ongoing monitoring and evaluation. IOM collected TB cohort data and Jordan NTP assessed their governorate and national diagnostic TB centers using CDC’s TB program evaluation tool for resource-limited and refugee settings [8]. For the cohort analysis, the objectives were to verify that at least 85 % of the new smear-positive TB cases were cured.
4. Increase TB awareness and knowledge of treatment services among Syrian refugees and their healthcare providers by information, education and communication (IEC) material for camp and non-camp settings, and
5. Support the development and implementation of national guidelines for management of latent TB infection.

Each of the objectives had activities with indicators and responsible partner(s) for collecting those indicators. These indicators were collected by IOM and NTP and shared with UNHCR and WHO.

#### Results of the public health strategy

**Fig. 1:**
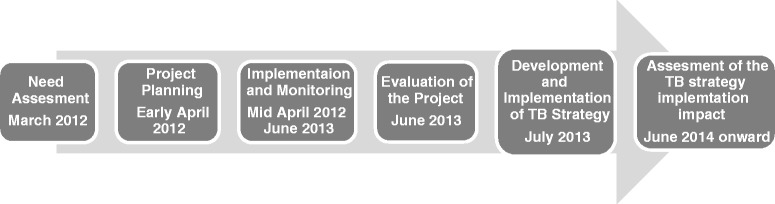
Timeline for implementing the TB strategy among Syrian refugees

The strategy was implemented in July 2013 (Fig. 1). Of the five strategic objectives, objective 4: increase TB awareness and knowledge of treatment services was to ensure that the Syrian refugees with suspected TB could enter the Jordan TB control program. This objective had activities for refugees and for healthcare providers. IEC material were developed for both groups to increase awareness of TB signs and symptoms with messaging for refugees that TB is curable and where to seek care if someone had symptoms suggestive of TB. IOM conducted hour-long TB awareness sessions in the camps and community with the aid of a local medical association and collected the numbers of refugees attending by age group and sex. This strategy for the first 6 months of 2014, resulted in almost 61,000 (10 % of total) Syrian refugees attending IOM conducted TB awareness sessions, with equal proportions of males and females (Table 1). As far as anecdotal accounts, several refugees presented to IOM reporting that they had TB signs and symptoms after reading the IEC materials or attending the awareness raising sessions, while others when asked did not report having any sign and symptoms presumably because of fear of stigma within their community with a smaller fear of being deported (exact numbers not captured).

**Table 1:** International Organization for Migration conducted TB awareness sessions, with TB screening and diagnosis performed in collaboration with the Jordan National TB Program among camp and non-camp Syrian refugees, 2014

Total	Total 2014	14-Jan	14-Feb	14-Mar	14-Apr	14-May	14-Jun
Awareness sessions	60,684	7,457	12,097	10,813	11,576	7,177	11,564
Male	30,465	3,942	6,242	5,395	5,860	3,584	5,442
%	50 %	53 %	52 %	50 %	51 %	50 %	47 %
Female	30,219	3,515	5,855	5,418	5,716	3,593	6,122
%	50 %	47 %	48 %	50 %	49 %	50 %	53 %
Screening	68,906	11,559	13,606	14,869	11,656	8,541	8,675
Male	33,160	5,667	6,482	7,065	5,604	4,214	4,128
%	48 %	49 %	48 %	48 %	48 %	49 %	48 %
Female	35,746	5,892	7,124	7,804	6,052	4,327	4,547
%	52 %	51 %	52 %	52 %	52 %	51 %	52 %
Under 15 years	30,886	4,792	6,787	7,449	5,654	4,015	2,189
%	45 %	41 %	50 %	50 %	49 %	47 %	25 %
Diagnosed	33	1	5	6	10	8	3
Male	19	0	3	2	8	4	2
%	58 %	0 %	60 %	33 %	80 %	50 %	67 %
Female	14	1	2	4	2	4	1
%	42 %	100 %	40 %	67 %	20 %	50 %	33 %

Second in importance was objective 1: increase TB screening, which offered Syrian refugees screening for TB symptoms, and if present, chest radiography and microscopic examinations, if indicated. With assistance of Jordan NTP and with UNHCR for newly arriving refugees, IOM screened almost 69,000 (11 % of total population) in first 6 months of 2014 (Table 1). Of those Syrians screened through June 2014, 45 % were 14 years of age or younger, which was comparable to the 45 % of the Syrian refugees aged 0 through 14 years derived from UNHCR regional demographic age structure [1]. Therefore, this suggests children were captured in the TB screening efforts.

For objective 3: maximize treatment success, IOM collected, maintained, and reported TB data among Syrian refugees. Examining the 2013-2014 TB cohort data for Syrian refugees on treatment, for the first three quarters (January-March 2013, April-June 2013, July-September 2013 followed for 9 months each), 91 %, 96 %, and 85 % had completed their anti-TB treatment (or been cured) for an overall completion rate of 91 % (Table 2), with one death and no failures. For the first quarter, there was a 9 % (two persons) treatment default rate; however, zero for the following two cohort periods. These initial two persons returned to Syria and were lost to follow-up. Subsequently, IOM increased its assistance to the refugees under treatment and emphasized with them the importance of treatment completion before travelling.

**Table 2:** Cohort results for 2014 of camp and non-camp Syrian refugee culture-confirmed patients who started anti-TB treatment 9 months prior

Tuberculosis cohort findings* by quarter, 2014	1st quarter % (n/N)	2nd quarter % (n/N)	3rd quarter % (n/N)
Cure/completion rate (without MDR†)	91 % (21/23)	96 % (23/24)	85 % (17/20)
Likely to complete treatment (without MDR)	91 % (21/23)	100 % (24/24)	95 % (19/20)
Death rate	0 % (0/0)	0 % (0/0)	5 % (1/20)
Default treatment rate	9 % (2/23)	0 % (0/0)	0 % (0/0)
Fail treatment rate	0 % (0/0)	0 % (0/0)	0 % (0/0)

*Source definitions: CDC. Understanding the TB cohort review process: instruction guide, 2006. [http://www.cdc.gov/tb/publications/guidestoolkits/cohort/method.htm]

†MDR, multiple-drug resistant

For objective 2: increase TB diagnosis, for the first 6 months of 2014, of the 33 Syrian refugees with culture confirmed active TB disease, one each was diagnosed in Syria and Kuwait arriving with treatment and three were diagnosed in Syria coming without any treatment; all five once in Jordan received testing and appropriate treatment begun or was continued. Only three were found to be highly infectious with sputum smears containing *Mycobacterium tuberculosis* bacilli identified microscopically (smear-positive), 18 (78.3 %) were sputum smear-negative, and two (6 %) were children under 5 years of age (Table 3). The high percentage of smear-negative TB cases was because a) some patients were partially treated in Syria before coming to Jordan but were considered new cases once in Jordan, and b) with the highly dynamic movement of refugees within Jordan and the fear of losing cases, NTP increased the clinical diagnosis of TB to include smear-negative TB cases who had three additional criteria: (i) cough for more than 3 weeks, (ii) no response to an non-TB antibiotics, and (iii) a chest radiograph compatible with TB.

**Table 3:** Culture-confirmed TB cases among camp and non-camp Syrian refugees, through June 2014

Type/site of tuberculosis	Case number
Pulmonary smear positive	3
Pulmonary smear negative	18
Pulmonary smear not available	2
Extra-pulmonary	10*
TOTAL	33

*2 among children less than 5 years of age

The public health strategy since initial development has identified TB cases among Syrian refugees at a higher rate (1.01 per 100,000 monthly or 12.17 per 100,000 for July 2013 through June 2014), than the pre-strategy period (0.73 per 100,000 monthly or 8.75 per 100,000 for 12 months), or approximately 40 % greater, using UNHCR population estimates (Table 4) [1]; more than double the Jordan TB incidence (5.8 per 100,000) for 2012 [10].

**Table 4:** Culture-confirmed TB cases identified among camp and non-camp Syrian refugees in Jordan by timing of the public health strategy, with rates based on population estimates from UNHCR*

	Pre-strategy 03/12-06/13	Strategy development and implementation 07/13-6/14
TB cases	56	74
No. refugees at period end*	480,000	608,000
Months of period	16	12
Cases per month	3.50	6.17
Cases per month per 100,000 Syrian population	0.73	1.01
Cases per 12 months per 100,000	8.75	12.17

*Source: United Nations High Commissioner for Refugees: Syria Regional Refugee Response [http://data.unhcr.org/syrianrefugees/regional.php]

## Discussion and evaluation

Rising TB prevalence rates have been observed among internally displaced persons within the Middle East and Eurasia regions. The Iraqi TB prevalence of 94/100,000 population in 1990, dropped to 62/100,000 in 2000, but rose to 74/100,000 in 2011 following years of armed conflict [2]. In Bosnia and Herzegovina, new TB cases increased 50 % over the wartime of the early 1990s [11]. After the independence of Georgia from the Soviet Union, both latent TB infection and active disease were common and screening, as currently offered in Jordan for Syrian refugees, was found to be a useful tool to find cases among that high prevalence population [12]. Additionally, a systematic review of crisis-affected populations (those experiencing displacement, armed conflict, or natural disasters) largely found elevated rates of TB notification and of TB prevalence [13]. These reported higher active TB rates probably developed over time and may be seen as the Syrian crisis approaches its fourth year.

In the meantime, internally displaced Syrians are living in a crisis situation. Living in a conflict zone may result in delayed TB treatment and increased self-treatment among TB patients as reported in other conflicts [14]. Drug-resistant TB has been found among resettling refugees from many settings who are arriving in such countries as the United Kingdom, Sweden and the United States [15–17]. Furthermore, interrupted TB treatment is a risk factor for developing MDR-TB, carrying substantial increased morbidity and mortality, and increased healthcare costs. Officials of the Jordan NTP noted that the monthly cost of MDR-TB medication is Jordanian Dinar (JOD) 2,000 versus JOD150 (over US$2,800 vs. US$210 monthly) for susceptible-TB medication; in addition, MDR-TB treatment is 24 months [18].

Another reason for higher prevalence rates among refugees may be because of increased detection. In the United States, resettled refugees compared with immigrants are offered free domestic health assessments [19]. This more active system may lead to the higher TB prevalence seen among refugees compared with other foreign-born persons. This active screening and case detection are probably at play in the Jordanian Public Health TB Strategy.

The main challenges faced during the implementation of the strategy, included

1. Syrian refugees are scattered in different governorates of Jordan outside of the camp setting, which made the screening process more challenging. IOM used a mobile radiograph vehicle and worked with NTP to conduct chest radiographs for suspected cases.
2. Some TB patients did not comply with the DOTS protocol and needed a close follow up to make sure they were taking their medications. IOM visited patients 5 times per week in the intensive phase and 3 times per week during the continuation phase.
3. Some of the extra-pulmonary TB patients suffered from other co-morbid medical conditions/complications, such as epileptic seizures, vertebral deformities, paralysis, urine incontinence, intestinal obstruction, etc. and required costly medical interventions. IOM tried to coordinate with other health providers that could provide appropriate assistance and interventions.
4. Inside the camps, the stigma issue was higher than outside the camps because of the overcrowded living conditions. IOM tried to ensure the privacy of the patients while raising awareness among refugees and host community members who could assist in overcoming the stigma.

Despite outlined challenges to TB control in refugee settings, the strength of Jordan TB control program offered a unique opportunity to improve refugee health, prevent new TB cases, and minimize the emergence of MDR-TB. There has been an almost 40 % increase in detected TB case rate among the Syrian refugees in Jordan since the TB strategy was implemented in July 2013 (while only a 25 % increase in the number of Syrian refugees). Tuberculosis is often an unrecognized disease, especially among children; therefore, providing screening for both children and adults may allow for detection and potentially at an earlier phase of the disease, understanding that diagnosis among children is more difficult than among adults [20]. Of those Syrians screened under the public health strategy, the proportions who were children were comparable with their proportion per UNHCR regional demographic age structure. Of the Syrian refugees with pulmonary TB, almost 80 % were sputum-smear negative potentially attesting to less risk of transmission of TB among the refugee as well as the host population.

However, no program is without limitations. Although, the TB strategy has screened over 10 % of the Syrian refugee population in the first 6 months of 2014, this screening needs to continue to find refugees who have been in the country since 2011. Furthermore, the calculated TB incidence of 12.2 per 100,000 per year for the strategy is less than the WHO 2012 estimated incidence rate of 18 (range 15-21) per 100,000 per year [10]. As mentioned before as the crisis continues to unfold, the incidence can only be expected to increase.

Additional limitations include the difficulties in securing a steady line of funding to continue the strategy over time. The CDC Evaluation Tool for Tuberculosis Programs in Resource-limited, Refugee and Post-Conflict Settings, version 2 has not been used at all governorate chest clinics because of limited human resource availability within the NTP. Although the contingency plans for movement to other countries for treatment were developed, they were not successfully implemented because cross-border communication with other national TB programs has not been sufficiently established. National TB program guidelines and practices are different in different countries. For example, some countries provide free follow-up laboratory testing, while others in the region do not provide such service. However, IOM in Jordan does work closely with its other missions in the region informing them when a refugee patient travels to their respective country. IOM-Jordan obtains the patient’s contact details and provides these to the respective mission; that mission then follows the patient until the completion of his/her treatment course. Finally, protocol for the treatment of latent TB infection still awaits development and implementation.

Given the WHO objective for any TB control program to cure at least 85 % of newly identified smear-positive TB cases, the Jordan program for Syrian refugees is performing to standards. These measures from TB awareness to screening and diagnosis matched with treatment activities specifically for Syrian refugees have ensured this level of treatment success and should be considered by neighboring countries.

## Conclusions

Jordan NTP, IOM, and UNHCR with technical assistance of WHO and CDC have developed a cohesive strategy to improve case detection and implement DOTS. This public health strategy was integrated into the Jordan NTP national strategy in December 2013. Initial assessment of this strategy has found that both Syrian children and adults are being diagnosed with TB at a high incidence rate and probably leading to less transmission of TB (only three sputum smear-positive patients) and less risk of drug-resistant TB. The public health TB control strategy needs continued support and strengthening to reach all Syrian refugees with potential TB, especially those who arrived in the early months of the crisis. However, this strategy attests to the importance of active screening of arriving refugees and needs to be considered in neighboring countries of the region such as Iraq, Lebanon, and Turkey. A successful TB strategy for Syrian refugees in Jordan has the potential to inform future treatment and control efforts for other regional countries impacted by the Syrian crisis.

## Abbreviations

CDC: US Centers for Disease Control and Prevention
DOTS: Directly observed therapy short-course
IEC: Information, education and communication
IOM: International Organization for Migration
JOD: Jordanian Dinar
MDR: Multidrug resistant
NTP: National Tuberculosis Program
TB: Tuberculosis
UNHCR: United Nations High Commissioner for Refugees
WHO: World Health Organization
